# A single-arm phase 2 study of abemaciclib in adult patients with recurrent grade 3 oligodendroglioma

**DOI:** 10.1093/noajnl/vdaf011

**Published:** 2025-01-17

**Authors:** Carson A Wills, Suyash Mohan, Ali Nabavizadeh, Thara Patel, Timothy Prior, Maikel Mansour, Emily McCoy, Shivani Shah, Natalie Angeloni, Meghan O’Neill, Suzanne Frangos, Caroline Blessing, Leah Coghlan, Eileen Maloney, E Paul Wileyto, Arati S Desai, Stephen J Bagley

**Affiliations:** Department of Medicine, University of Pennsylvania Perelman School of Medicine, Philadelphia, Pennsylvania, USA; Department of Radiology, University of Pennsylvania Perelman School of Medicine, Philadelphia, Pennsylvania, USA; Department of Radiology, University of Pennsylvania Perelman School of Medicine, Philadelphia, Pennsylvania, USA; Department of Neurosurgery, University of Pennsylvania Perelman School of Medicine, Philadelphia, Pennsylvania, USA; Department of Neurosurgery, University of Pennsylvania Perelman School of Medicine, Philadelphia, Pennsylvania, USA; Department of Neurosurgery, University of Pennsylvania Perelman School of Medicine, Philadelphia, Pennsylvania, USA; Department of Neurosurgery, University of Pennsylvania Perelman School of Medicine, Philadelphia, Pennsylvania, USA; Department of Neurosurgery, University of Pennsylvania Perelman School of Medicine, Philadelphia, Pennsylvania, USA; Department of Neurosurgery, University of Pennsylvania Perelman School of Medicine, Philadelphia, Pennsylvania, USA; Abramson Cancer Center, University of Pennsylvania, Philadelphia, Pennsylvania, USA; Department of Neurosurgery, University of Pennsylvania Perelman School of Medicine, Philadelphia, Pennsylvania, USA; Department of Neurosurgery, University of Pennsylvania Perelman School of Medicine, Philadelphia, Pennsylvania, USA; Department of Neurosurgery, University of Pennsylvania Perelman School of Medicine, Philadelphia, Pennsylvania, USA; Department of Neurosurgery, University of Pennsylvania Perelman School of Medicine, Philadelphia, Pennsylvania, USA; Abramson Cancer Center, University of Pennsylvania, Philadelphia, Pennsylvania, USA; Division of Hematology/Oncology, Department of Medicine, University of Pennsylvania Perelman School of Medicine, Philadelphia, Pennsylvania, USA; Abramson Cancer Center, University of Pennsylvania, Philadelphia, Pennsylvania, USA; Division of Hematology/Oncology, Department of Medicine, University of Pennsylvania Perelman School of Medicine, Philadelphia, Pennsylvania, USA; Abramson Cancer Center, University of Pennsylvania, Philadelphia, Pennsylvania, USA

**Keywords:** abemaciclib, CDK4/6 inhibition, cell cycle inhibition, IDH-mutant glioma, oligodendroglioma

## Abstract

**Background:**

Novel treatments are needed for oligodendroglioma that has recurred following radiotherapy (RT) and chemotherapy. The cyclin D1–CDK4 axis is frequently dysregulated in oligodendroglioma. Abemaciclib is a selective CDK4/6 inhibitor that achieves pharmacologically relevant concentrations in brain tumor tissue.

**Methods:**

We conducted a single-arm, phase 2 trial evaluating the efficacy of abemaciclib in patients with recurrent oligodendroglioma, isocitrate dehydrogenase (IDH)-mutant and 1p/19q-codeleted, WHO grade 3, following prior RT and ≥1 line of alkylating chemotherapy. Patients received abemaciclib 200 mg twice daily. The primary endpoint was progression-free survival at 6 months (PFS-6).

**Results:**

Ten patients were enrolled. The most common treatment-related adverse event was grade 1–2 diarrhea, occurring in all patients. Five patients (50%) were alive and progression-free at 6 months, below the minimum required (80%) to meet the primary endpoint. In patients with enhancing tumor (*n* = 9), best response was partial response in 2 patients (objective radiographic response = 22.2%; duration of response [DOR] 13.1 and 7.7 months), stable disease (SD) in 3 patients (33.3%; duration of SD 17.0, 6.7, and 2.5 months), progressive disease in 3 patients (33.3%), and nonevaluable in 1 patient (11.1%). The patient with nonenhancing tumor showed SD lasting 10.2 months. Median PFS was 7.7 months (95% CI, 1.7–13.1 months); median overall survival was not reached (median follow-up 17 months).

**Conclusions:**

The efficacy of abemaciclib in recurrent grade 3 oligodendroglioma was inadequate to warrant further evaluation as monotherapy in unselected patients. However, given the objective responses and durable disease control observed in a subset of patients, further studies are warranted to identify subgroups that may benefit.

## Lay Summary

Oligodendroglioma is a common brain tumor that is difficult to treat and often grows back after radiation and chemotherapy. Abemaciclib is a drug that targets proteins that help some tumors grow. The authors of this study wanted to see if abemaciclib could help control tumor growth in patients with oligodendrogliomas that grew after initial treatment with radiation and chemotherapy. To do this, they tested the drug in a clinical trial of 10 patients. Overall, the drug seemed safe. After 6 months, 5 patients (50%) were alive and had no tumor growth, which was lower expected rate of 80%. Some patients even had partial shrinkage of the tumor.

**Key Points**

- Abemaciclib was well tolerated with grade 1–2 diarrhea as the most common adverse event.
- PFS-6 was achieved in 5 of 10 patients (50%); the study did not meet its primary endpoint.
- Two patients experienced partial response to treatment, suggesting a potential benefit in select patients.

**Importance of the Study**

Oligodendroglioma is a rare and incurable primary brain tumor. Prospective clinical trials in this specific patient population, as molecularly defined by modern WHO criteria, are uncommon. Although oligodendroglioma initially responds well to radiation and alkylating chemotherapy, all patients inevitably relapse and have limited treatment options. Here we present a single-arm, phase 2 study of abemaciclib, a selective small molecule inhibitor of CDK4 and CDK6 that achieves therapeutic concentrations in the brain, in patients with recurrent grade 3 oligodendroglioma. Although the study did not meet its primary endpoint for efficacy (PFS-6), the treatment was well tolerated and resulted in objective radiographic response or durable stable disease in a subset of patients. Taken together, our results suggest that further investigation is needed to identify the subset of oligodendroglioma patients that may benefit from CDK4/6 inhibition and to determine if combinations involving abemaciclib may provide improved efficacy.

Oligodendroglioma, genetically defined by mutation in the metabolic enzyme isocitrate dehydrogenase 1 (*IDH1*) or 2 (*IDH2*) gene and an unbalanced translocation between chromosomes 1 and 19 (1p/19q-codeleted),^1,2^ is a rare primary brain tumor diagnosed in approximately ~1000 patients each year in the United States. The World Health Organization (WHO) grading system further characterizes these tumors as low-grade (grade 2) or high-grade (grade 3) based on histopathologic features.^1^ As with other diffuse gliomas, surgery is not curative, and radiation therapy (RT) and alkylating chemotherapy (either temozolomide and/or procarbazine, CCNU, and vincristine [PCV]) are eventually required in all patients.^1,3,4^ While IDH 1/2 inhibition with vorasidenib has recently demonstrated efficacy in patients with newly diagnosed grade 2 oligodendroglioma, this therapy only delays progression of disease and the need for RT and chemotherapy.^5^ Overall, despite being initially slow-growing and highly sensitive to treatment, recurrence of oligodendroglioma following RT and alkylating chemotherapy is inevitable and typically occurs with high-grade features and more aggressive biology.^6^ Salvage therapy options for recurrent disease include repeat surgery, re-irradiation, and chemotherapy (either PCV or temozolomide, depending on what the patient received for first-line therapy). However, duration of disease control is generally shorter compared with treatment at first diagnosis, and nearly all patients will ultimately succumb to the disease. Thus, there is critical need for development of novel and effective therapeutic options.

Approximately 50%–70% of oligodendrogliomas harbor a mutation in the Capicua (*CIC*) tumor-suppressor gene,^7^ which encodes a high-mobility group box transcriptional repressor that functions as a key regulator of mitogen-activated protein kinase pathway activation and cellular proliferation.^8,9^ CIC has also been shown to bind to the cyclin D1 (*CCND1*) gene, an important regulatory subunit of cyclin-dependent kinase (CDK) 4 and CDK6 that promotes cell cycle progression from G1 to S phase and that is frequently upregulated in recurrent gliomas.^8^ In a mouse model of oligodendroglioma, loss of cyclin D1 and CDK4 suppressed tumor progression, highlighting the role of the CD4/6-cyclin D1 axis in this disease and suggesting a viable pharmacologic target.^10^ In patients with oligodendroglioma, *CIC* mutation is associated with worse prognosis.^11,12^ In addition to a high rate of *CIC* mutations, recurrent high-grade oligodendrogliomas also exhibit frequent loss of cyclin-dependent kinase inhibitor 2A (*CDKN2A*), a tumor-suppressor gene located at 9p21 that encodes p16^(INK4A)^ and p14^(ARF)^ proteins, with homozygous deletion in approximately one-third of cases and hypermethylation in more than half.^13,14^ Previous studies have demonstrated that grade 3 oligodendrogliomas (defined by the presence of *IDH* mutation and 1p/19q codeletion) harboring *CDKN2A* homozygous deletion have significantly worse prognosis compared to those lacking this alteration.^15^ Taken together, these data provide rationale for therapeutic targeting of the CDK/Rb pathway in patients with recurrent oligodendroglioma.

Abemaciclib is a selective small molecule inhibitor of CDK4 and CDK6 that is approved by the U.S. Food and Drug Administration (FDA) for the treatment of hormone receptor-positive, human epidermal growth factor receptor 2 (HER2)-negative advanced or metastatic breast cancer. Compared to other clinically available dual CDK4/6 inhibitors, abemaciclib exhibits increased blood–brain barrier penetration and CDK4 selectivity and has been shown to achieve therapeutic concentrations in brain tissue.^16,17^ Notably, patient-derived IDH-mutant glioma cells, including those derived from oligodendroglioma, were recently shown to exhibit decreases in cell viability and proliferation when treated with abemaciclib in vitro, and abemaciclib improved survival in orthotopically implanted IDH-mutant glioma xenograft models.^18^

To evaluate for an efficacy signal in the treatment of recurrent oligodendroglioma, we conducted a single-center, single-arm, open-label phase 2 clinical trial of abemaciclib monotherapy in patients with recurrent, IDH-mutant and 1p/19q-codeleted, WHO grade 3 disease who had previously received RT and at least 1 line of alkylating chemotherapy.

## Methods

### Patient Eligibility

Patients aged 18 years or older were recruited from the Medical Oncology, Radiation Oncology, and Neurosurgery practices at the University of Pennsylvania Health System between November 2019 and August 2022. Patients with recurrent oligodendroglioma, defined by the presence of IDH mutation and 1p19q codeletion, were eligible for the study. Loss of 1p/19q was confirmed through fluorescent in situ hybridization, genomic sequencing, or methylomic analysis performed in a Clinical Laboratory Improvement Act-certified laboratory. Tumors could have been either grade 2 or grade 3 at original diagnosis but must have progressed or recurred following RT and at least 1 line of alkylating chemotherapy. Measurable disease was required (at least 1 cm × 1 cm enhancing tumor in patients with enhancing disease, or at least 1 cm × 1 cm T2/FLAIR disease in patients with nonenhancing disease). Patients may have had treatment for an unlimited number of relapses. Additional *key inclusion criteria* included Karnofsky performance status **≥**60, life expectancy >3 months, and adequate end-organ function. *Key exclusion criteria* included prior treatment with a CDK4/6 inhibitor, treatment with enzyme-inducing anti-seizure medications, and concurrent treatment with any other anticancer agents.

### Study Design

This was a single-center, single-arm, open-label phase 2 study. Abemaciclib tablets were supplied by Eli Lilly for investigational use. Patients were treated with abemaciclib 200 mg by mouth once every 12 h on continuous 28-day treatment cycles until radiographic evidence of progression, unacceptable toxicity, patient withdrawal of consent, or death. This dose is the previously defined maximum tolerated dose and recommended phase 2 dose for single-agent abemaciclib, as well as the FDA-approved dose for use as monotherapy in adults with hormone receptor (HR)-positive, human epidermal growth factor receptor 2 (HER2)-negative advanced or metastatic breast cancer with disease progression following endocrine therapy and chemotherapy.^16^ Dose suspensions and reductions for hematologic and nonhematologic toxicities were allowed according to the drug’s FDA label for breast cancer. The primary endpoint was progression-free survival status at 6 months (PFS-6) following trial registration, that is, a binary endpoint. Secondary endpoints included evaluating the safety and tolerability of abemaciclib (as measured by the frequency, duration, and severity of adverse events [AEs]), objective radiographic response (ORR), PFS, and overall survival (OS). This study (ClinicalTrials.gov identifier NCT03969706) was approved by the Institutional Review Board of the University of Pennsylvania, and written informed consent was obtained from all subjects.

### Assessments

An AE was defined as any symptom, sign, illness, or experience that developed or worsened in severity during the course of the study, whether or not considered drug-related. AEs were graded according to the NCI Common Terminology Criteria for Adverse Events version 5.0. A treatment-related AE (TRAE) was any treatment-emergent event that the investigator-assessed had at least a reasonable possibility of having a causal relationship with the study drug. Routine laboratory tests were performed locally and included assessments of hematology parameters and serum chemistries (at screening, days 1 and 15 of cycle 1 and cycle 2, and day 1 of every cycle thereafter).

MRI of the brain with and without gadolinium was performed at the time of screening and at least once every 8 weeks during treatment. In patients with enhancing tumors, modified Response Assessment in Neuro-Oncology (mRANO) criteria were used to assess tumor response and progression.^19^ For patients with nonenhancing tumors, RANO low-grade glioma criteria were used.^20^

### Statistical Design and Analysis

Patients were enrolled in a single-stage design. The primary endpoint was PFS-6. The alternative hypothesis was that the proportion of patients without an event (progression of disease or death due to disease or toxicity) at 6 months from the time of study enrollment would be 80% or higher. This was tested against a null hypothesis of 50% of subjects without an event at 6 months, which was based on the historical PFS-6 rate in previous trials of systemic therapies for oligodendroglioma that has recurred after RT and alkylating chemotherapy.^21–23^ A PFS-based primary endpoint was chosen, rather than a response rate primary endpoint, due to both (i) the predominantly cytostatic nature of abemaciclib,^24^ and (ii) the fact that a therapy that results in prolonged stable disease, even in the absence of tumor regression, would be of clinical value.

With a sample size of 10 patients, the power to detect the difference between the null and alternative hypotheses was 82%, with a probability of type I error (alpha) of 0.10 (1-sided). The study would be considered positive if 8 or greater subjects were event-free at 6 months. Median PFS and median OS were estimated using the Kaplan–Meier method, with patients censored at their last known date alive. Median follow-up time was calculated using the reverse Kaplan–Meier method. Sample size calculation was performed with PASS 2022 Power Analysis and Sample Size Software (NCSS, LLC). All other statistical analyses were performed using Stata, version 14.2 (StataCorp).

## Results

### Patient Characteristics

Ten patients were enrolled between November 2019 and August 2022. Baseline patient characteristics are displayed in Table 1. The median age at the time of enrollment was 50 years, with a median of 14.3 years since original diagnosis. All patients had previously undergone RT and treatment with temozolomide, with a median of 6.7 months since last systemic therapy. Five of 10 (50%) patients had previously received 3 or more lines of any systemic therapy, including 4 of 10 (40%) treated with at least 2 lines of alkylating chemotherapy and 3 of 10 (30%) previously treated with an IDH inhibitor (Supplementary Table 1). At the time of enrollment, 9 of 10 (90%) patients demonstrated progressive enhancing tumors, while 1 of 10 (10%) had a progressive nonenhancing tumor. Multifocal disease was observed in 8 of 10 (80%) patients at the time of enrollment.

**Table 1.** Patient Characteristics (*N* = 10)

Patient characteristics	
Age at enrollment, years (median, IQR)	50 (44, 55)
Female gender, *n* (%)	5 (50)
Karnofsky performance status **≥**90	9 (90)
Tumor grade 3 (WHO) at screening	10 (100)
Enhancing tumor at screening	9 (90)
Multifocality at enrollment	8 (80)
Median time since original diagnosis, years (range)	14.3 (3.9, 19.7)
Median time since last systemic therapy, months (range)	6.7 (0.4, 132)
Prior radiotherapy	10 (100)
No. of prior lines of systemic therapy	
1	5 (50)
2	0
3	1 (10)
**≥**4	4 (40)
Prior temozolomide chemotherapy	10 (100)
No. or prior lines of alkylating chemotherapy	
1	6 (60)
2	4 (40)

Abbreviations: IQR, interquartile range; WHO, World Health Organization.

### Efficacy

**Figure 1.**
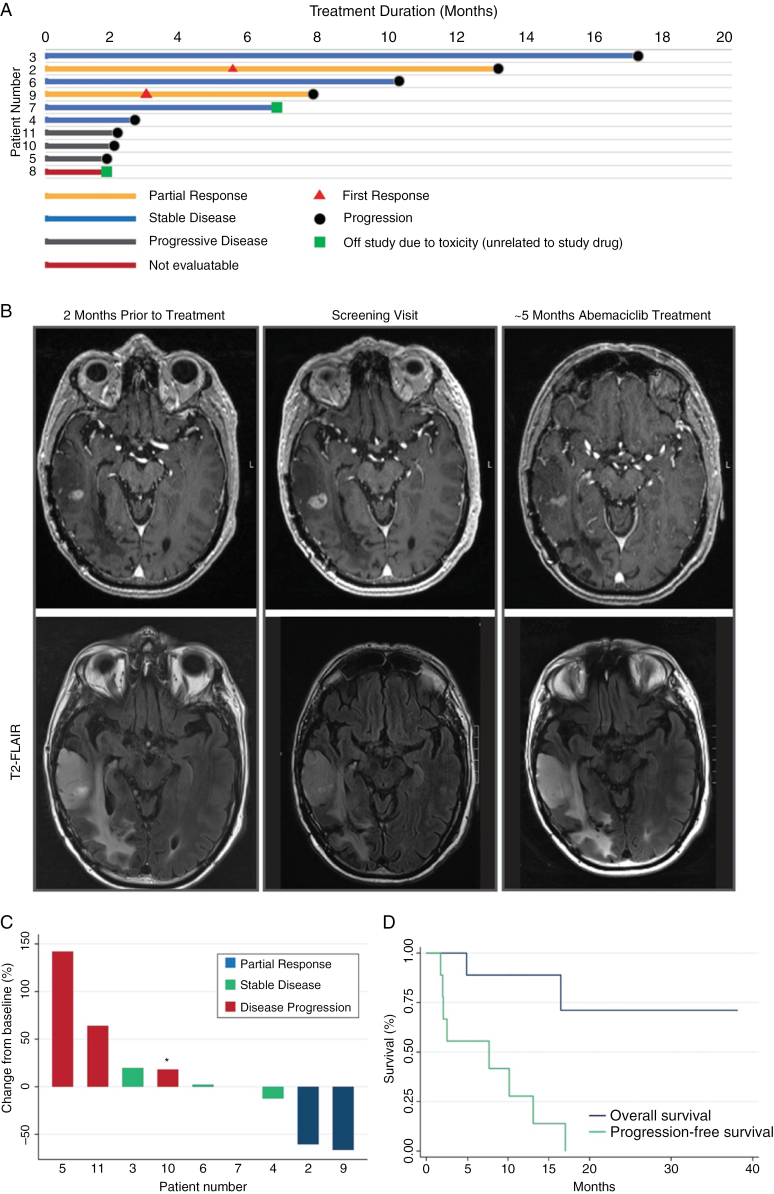
(A) Swimmer’s plot of response to treatment. Patients in rows 1 and 2 had tumors characterized by both MSH6-mutation and temozolomide-induced hypermutation. *N* = 10. (B) MRI brain of patient 2 months prior to screening (left), at the time of screening visit (center), and after approximately 5 months of treatment with abemaciclib (right), demonstrating partial response to treatment. T2/FLAIR signal (bottom) at corresponding time points. (C) Waterfall plot demonstrating response to treatment. *Patient with clinical progression despite <25% increase from baseline. *N* = 9. (D) Progression-free (PFS) and overall survival (OS) in months. Median PFS = 7.7 months (95% CI, 1.7–13.1 months). PFS-6 months = 50%. Median OS = not reached (NR) (95% CI, 4.9 months–NR).

Five of 10 patients were alive and progression-free at 6 months from the time of enrollment (PFS-6 = 50%), below the minimum threshold (PFS-6 = 80%) required for the study to meet its primary endpoint (Figure 1A). A representative response with stable T2/FLAIR signal is displayed in Figure 1B. Best response achieved for each patient is displayed in Table 2, with a waterfall plot showing percentage changes in patients’ tumors from baseline in Figure 1C. Of the participants with an enhancing tumor at baseline (*n* = 9), 2 achieved a partial response by mRANO criteria (ORR = 22%). In the 2 responding patients, duration of response was 13.1 and 7.7 months, with onset of response first at 5 and 3 months, respectively (Figure 1A). The most recent RT courses were remote for both responders (8 and 4 years prior to study enrollment, respectively), and both responders had elevated relative cerebral blood volume in the tumor lesions on advanced MRI at the time of study enrollment. Three patients with enhancing tumors had stable disease (33.3%) over the course of treatment, with a duration of 17.0, 6.7, and 2.5 months, respectively (Figure 1A). Three patients experienced disease progression (33.3%), with 1 patient experiencing clinical progression despite a <25% increase in tumor from baseline (Figure 1C). One patient with a nonenhancing tumor at baseline showed stable disease for 10.2 months. Disease progression could not be evaluated in 1 patient (11.1%), who was removed from the study after 2 months due to diagnosis of a new, separate malignancy that required immediate therapy. The median PFS for all patients enrolled in this study was 7.7 months (95% CI, 1.7–13.1 months) (Figure 1D). At the time of data cutoff for this analysis (median follow-up 17 months), median OS was not yet reached, as 7 of 10 (70%) patients were alive.

**Table 2.** Investigator-Reported Best Overall Response

Response	Modified RANO Criteria (*n* = 9)	ANO LGG Criteria (*n* = 1)
Best overall response, no. (%)		
Complete response	0	0
Partial response	2 (22.2)	0
Minor response	-	0
Stable disease	3 (33.3)	1 (100)
Progressive disease	3 (33.3)	0
Not evaluable	1 (11.1)	0

Abbreviations: ANO LGG, Assessment in Neuro-Oncology low-grade glioma; RANO, Response Assessment in Neuro-Oncology.

Of the patients who achieved the PFS-6 endpoint (*n* = 5), 4 patients (80%) had only received 1 line of prior systemic therapy (temozolomide in all cases), while 1 patient (20%) had received 5 prior lines of systemic therapy, including an IDH1/2 inhibitor and 1 line of alkylating chemotherapy with temozolomide (Supplementary Table 1). Previous targeted next-generation sequencing (NGS) results from archival tumor were available for 8 of 10 (80%) patients. *CIC* mutation was present in 4 of these 8 (50%) patients’ tumors, although was absent in 3 of the 4 patients who achieved PFS-6 and had targeted NGS results available. Of the 8 patients with available NGS results, only 5 had tumors tested for *CDKN2A/B* homozygous deletion, and only 1 of the 5 (20%) patients tested was positive for *CDKN2A/B* homozygous deletion. Of the patients who experienced partial response, 1 tested negative for *CDK2A/B* homozygous deletion, while the other did not undergo tumor sequencing. The patient who was positive for homozygous deletion of *CDKN2A/B* came off study 2 months into treatment due to an AE, unrelated to study drug. Notably, post-temozolomide hypermutation phenotype was observed in 2 of the 8 patients (25%), with tumor mutational burden (TMB) of 381.6 mutations per megabase and 87.5 mutations per megabase, respectively. Both of these patients achieved partial responses to abemaciclib.

### Safety and Toxicity

All patients treated with abemaciclib in this study experienced at least 1 TRAE (Table 3). The most common TRAE was grade 1–2 diarrhea, which occurred in all 10 patients. Grade 3–4 TRAEs included grade 4 thrombocytopenia (*n* = 2), grade 3 neutropenia (*n* = 1), grade 3 fatigue (*n* = 2), and a grade 3 alanine aminotransferase increase (*n* = 1). TRAEs leading to dose reduction were seen in 2 (20%) patients for grade 3 thrombocytopenia and grade 3 fatigue. TRAEs leading to dose interruption occurred in 7 (70%) patients, including for grade 3–4 thrombocytopenia (*n* = 2, 20%), grade 3 fatigue (*n* = 1, 10%), grade 3 neutropenia (*n* = 1, 10%), grade 2 abdominal pain (*n* = 1, 10%), grade 3 infection with COVID-19 (*n* = 1, 10%), and grade 2 diarrhea (*n* = 1, 10%).

**Table 3.** Treatment-Related Adverse Events

Event	Any Grade, No. (%)	Grade ≥3, No. (%)
Nonhematologic		
Sore throat	1 (10)	0
Mucositis	1 (10)	0
Constipation	2 (20)	0
Diarrhea	10 (100)	0
Nausea	3 (30)	0
Vomiting	1 (10)	0
Abdominal pain	2 (20)	0
Dysgeusia	2 (20)	0
Flatulence	2 (20)	0
Belching	1 (10)	0
Nail changes	1 (10)	0
Alopecia	2 (20)	0
Change in urine odor	1 (10)	0
Creatinine increased	3 (30)	0
Hypernatremia	1 (10)	0
Periorbital edema	1 (10)	0
Edema in limbs	1 (10)	0
Fatigue	4 (40)	2 (20)
Generalized weakness	1 (10)	1 (10)
Alanine aminotransferase increased	1 (10)	1 (10)
Aspartate aminotransferase increased	1 (10)	0
Insomnia	2 (20)	0
Cognitive disturbance	1 (10)	0
Irritability	1 (10)	0

## Discussion

In this single-center, single-arm phase 2 trial, abemaciclib monotherapy did not demonstrate adequate efficacy to warrant further clinical evaluation in unselected patients with recurrent grade 3 oligodendroglioma. However, treatment with abemaciclib resulted in meaningful, durable disease control (response or stable disease lasting at least 6 months) in 5 of the 10 patients treated, suggesting that there is a subset of patients with recurrent oligodendroglioma who may benefit with this therapy. This efficacy signal in select patients is noteworthy given that abemaciclib was well tolerated with only low-grade diarrhea and transient myelosuppression as the most common TRAEs in this study, comparing favorably relative to standard treatment options for recurrent oligodendroglioma (ie, additional cytotoxic chemotherapy and/or re-irradiation). Overall, the efficacy data from this trial suggest that further studies are needed to identify the subgroup(s) of patients with oligodendroglioma who may have tumors sensitive to CDK4/6 inhibition, and that novel therapeutic combinations incorporating CDK4/6 inhibition should be considered for preclinical and eventual clinical evaluation in this disease.

Given the strong biologic rationale and supporting preclinical data for evaluating CDK4/6 inhibition in patients with oligodendroglioma,^8,10,18^ a previous phase 2 clinical trial evaluated palbociclib, the first selective CDK4/6 inhibitor to receive FDA approval, in patients with recurrent retinoblastoma-positive anaplastic oligodendroglioma (AO).^25^ In that study, Sepulveda-Sanchez et al. enrolled 34 patients with AO that had progressed despite prior RT and chemotherapy and who had conserved retinoblastoma protein (pRb) expression in the tumor by immunohistochemistry. Patients were treated with palbociclib (125 mg/day) for 3 weeks on and 1 week off, with the primary endpoint of PFS-6. The study was stopped early due to lack of efficacy, with 74% of evaluable patients progressing within 6 months. Median PFS was 2.8 months, and median OS was 32.1 months. There were no objective radiographic responses. One potential reason for the limited efficacy observed in that trial may be the lower blood–brain barrier penetration of palbociclib relative to abemaciclib, although this cannot be the only reason, given that our study also did not meet its primary endpoint.

Although subgroup analyses must be interpreted with caution given the small sample size, it is noteworthy that the 2 patients who achieved a partial response to therapy in this study had hypermutated tumors at the time of trial enrollment, with markedly elevated TMB. As this hypermutator phenotype has been previously shown to be related to the development of mismatch repair (MMR) defects after treatment with temozolomide and is associated with aggressive clinical behavior and poor patient survival,^26^ responses to any systemic therapy in this population are notable. Moreover, recent preclinical studies suggest that CDK4/6 blockade may be particularly effective in MMR-deficient (dMMR) tumors, in part due to its immunomodulatory potential.^27^ Additional studies are needed to determine if dMMR/hypermutator recurrent oligodendrogliomas may benefit from CDK4/6 inhibition. It is also apparent that less heavily pretreated patients benefited more from abemaciclib than those who had received multiple lines of prior systemic therapy, as 4 of 5 patients who achieved the PFS-6 endpoint had only received 1 line of prior systemic therapy (temozolomide in all cases).

Although recurrent grade 3 oligodendroglioma represents a rare disease population, our study is limited by the small sample size. While the study was adequately powered to detect the difference between the null and alternative hypotheses for PFS-6, it is nonetheless difficult to draw firm conclusions regarding efficacy with only 10 patients treated. Other study limitations include its single-center, single-arm design, which introduces the possibility of selection bias, as well as the lack of central imaging review and the heterogeneity of the study population, including differing number and types of prior therapies and a wide range of time elapsed since original diagnosis and last systemic therapy. Lastly, we cannot completely rule out that the radiographic progression at time of enrollment in the 2 patients experiencing partial response to abemaciclib was related to treatment effect as opposed to true neoplastic progression.^28^ However, the long time periods elapsed between last RT and study enrollment (8 and 4 years, respectively) in these patients, as well as the presence of elevated relative cerebral blood volume noted in areas of enhancing tumor on advanced imaging at time of enrollment, argue that these patients had viable neoplastic progression entering the trial and likely experienced real responses to abemaciclib.

Collectively, these results show that the brain-penetrant CDK4/6 inhibitor abemaciclib was generally well tolerated in patients with recurrent grade 3 oligodendroglioma, but did not demonstrate adequate efficacy to warrant further investigation as monotherapy in this population. Notably, a subset of patients clearly experienced clinical benefit from abemaciclib with partial tumor responses and durable stable disease. Blood and tumor-based correlative analyses from this trial are ongoing to understand why some tumors were sensitive to abemaciclib and others resistant. Additional preclinical studies are also warranted to explore potential therapeutic combinations utilizing CDK4/6 inhibition alongside other therapies in IDH-mutant glioma.

## Supplementary Material

vdaf011_suppl_Supplementary_Table

## Data Availability

All data analyzed will be made available upon reasonable request.
