# Supplementary material for: A single-arm phase 2 study of abemaciclib in adult patients with recurrent grade 3 oligodendroglioma
Source: Neurooncol Adv. 2025 Jan 17;7(1):vdaf011. doi: 10.1093/noajnl/vdaf011 (PMC11815343; doi:10.1093/noajnl/vdaf011)
Supplement: vdaf011_suppl_Supplementary_Table [file vdaf011_suppl_Supplementary_Table.docx]

**Supplemental Table 1.** Systemic therapy prior to enrollment in patients receiving at least 3 prior lines of systemic therapy

| **Patient** | **Prior Systemic Therapy** |
| --- | --- |
| 4 | 1. Temozolomide 2. Isotretinoin 3. Lomustine 4. Bevacizumab |
| 5 | 1. Temozolomide 2. Procarbazine/Lomustine 3. Vorasidenib, Olaparib + Temozolomide |
| 8 | 1. Temozolomide 2. Procarbazine/Lomustine/Vincristine 3. Nivolumab/Ipilimumab |
| 9 | 1. Temozolomide 2. Temozolomide + Hydroxyurea 3. Vorasidenib 4. Oncolytic viral Therapy rQNestin 5. Olaparib |
| 10 | 1. Temozolomide 2. Lomustine 3. Vorasidenib 4. Olaparib + Temozolomide |
